# Supplementary material for: Development of the Motor Periphery is the Rate-Limiting Step in the Ontogeny of the Vestibulo-ocular Reflex
Source: bioRxiv. 2024 May 17:2024.05.17.594732. Preprint. [Version 1] doi: 10.1101/2024.05.17.594732 (PMC11118585; doi:10.1101/2024.05.17.594732)
Supplement: 1 [file NIHPP2024.05.17.594732V1-supplement-1.pdf]

bioRxiv preprint doi: https://doi.org/10.1101/2024.05.17.594732; this version posted May 17, 2024. The copyright holder for this preprint (which was not certified by peer review) is the author/funder, who has granted bioRxiv a license to display the preprint in perpetuity. It is made available under aCC-BY 4.0 International license.

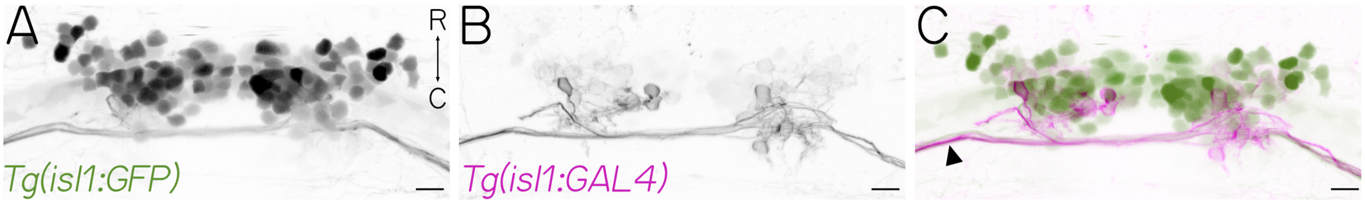

Figure S1: Sparse labeling of superior oblique extraocular motor neurons.
(A-C) Dorso-ventral maximum intensity projection of the extraocular motor neurons of cranial nuclei nIII and nIV visualized in 4 dpf fish using the triple transgenic line *Tg(isl1:GFP)* (A, green) crossed to *Tg(isl1:GAL4);Tg(UAS:KillerRed)* (B, magenta). Scale bar = 10μm.

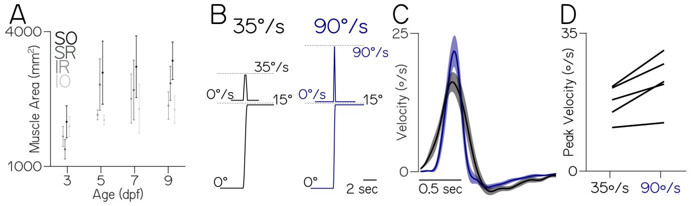

Figure S2: Stronger tilt stimulation evokes faster eye rotations before gaze stabilization behavior is fully mature
(A) Mean area (mm^2) ± SD in all four extraocular muscles from Figures 4H-I. **(B)** Schematic of our standard (left, black) table-tilt stimulus and a faster (right, blue) variant, as described in Figure 1A. Fish are stepped upwards to a a +15° (nose-up) step, held for 7.5 sec, then returned. In black, the peak velocity is 35°/sec and the peak acceleration is 150°/sec. In blue, the peak velocity is 90°/sec and the peak acceleration is 600°/sec. Traces derived from generated stimulus steps. **(C)** Eye velocity traces ± SEM of five fish at 5 dpf stepped using normal (black) or fast (blue) stimuli. **(D)** Mean peak eye velocity of 5 dpf fish for normal (black) and fast (blue) tilts. Peak eye velocities are significantly larger after fast tilts ($p_{t\text{-}test}$ = 0.016, N=5).

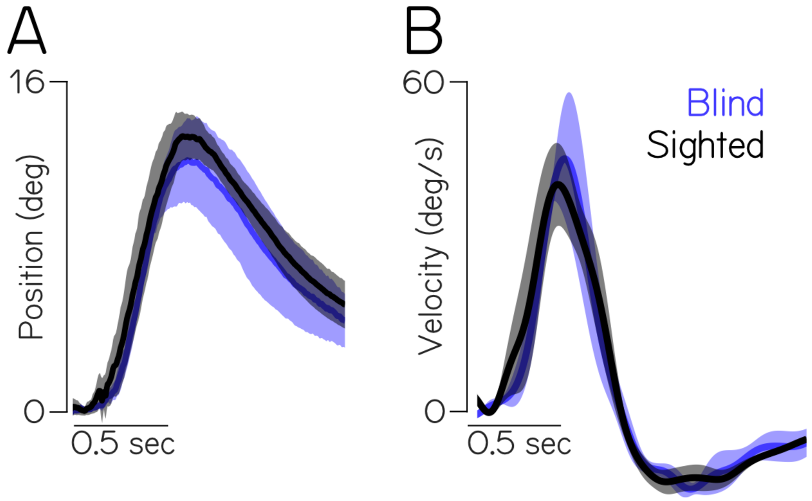

Figure S3: Congenitally blind fish achieve comparable gaze stabilization to sighted siblings
(A) Eye position traces ± SEM of six fish morphologically aged approximately 9 dpf stepped to +15°at peak velocity 35°/sec. Congenitally blind fish traces in blue (n = 3), sighted siblings in black (n = 3). **(B)** Eye velocity traces ± SEM of the same fish in panel A stepped to +15°at peak velocity 35°/sec.
